# Neuroplasticity and Repair in Rodent Neurotoxic Models of Spinal Motoneuron Disease

**DOI:** 10.1155/2016/2769735

**Published:** 2016-01-03

**Authors:** Rosario Gulino

**Affiliations:** Department of Biomedical and Biotechnological Sciences, Physiology Section, University of Catania, Via Santa Sofia 64, 95125 Catania, Italy

## Abstract

Retrogradely transported toxins are widely used to set up protocols for selective lesioning of the nervous system. These methods could be collectively named “molecular neurosurgery” because they are able to destroy specific types of neurons by using targeted neurotoxins. Lectins such as ricin, volkensin, or modeccin and neuropeptide- or antibody-conjugated saporin represent the most effective toxins used for neuronal lesioning. Some of these specific neurotoxins could be used to induce selective depletion of spinal motoneurons. In this review, we extensively describe two rodent models of motoneuron degeneration induced by volkensin or cholera toxin-B saporin. In particular, we focus on the possible experimental use of these models to mimic neurodegenerative diseases, to dissect the molecular mechanisms of neuroplastic changes underlying the spontaneous functional recovery after motoneuron death, and finally to test different strategies of neural repair. The potential clinical applications of these approaches are also discussed.

## 1. Introduction

Motoneuron loss is the common feature of several neurodegenerative diseases, as well as mechanical injuries affecting the spinal cord (SC). Among neurodegenerative diseases, amyotrophic lateral sclerosis (ALS) and spinal muscular atrophy (SMA) represent the most common diseases affecting spinal and brainstem motoneurons. ALS has enormous impact on the quality of life [1–3]. This disease affects mainly the lower motoneurons within the SC and brainstem, but the pyramidal neurons located in the motor cortex are also frequently damaged. This results in progressive muscle atrophy and spasticity, which ultimately cause death due to respiratory dysfunction [1, 4]. ALS is a heterogeneous disease complex that could be subdivided into two main groups: familial ALS (fALS), which accounts for only 10% of patients, and the more frequent form with no family history, affecting the remaining 90% of ALS patients, namely, the sporadic ALS (sALS) [1, 4]. The molecular mechanisms of ALS pathogenesis remain far to be fully understood and appear extremely heterogeneous. However, a number of gene mutations have been found in fALS patients, including a missense mutation in the SOD1 gene, encoding for superoxide dismutase 1 protein, which is the most frequent gene mutation found in fALS. More recently, aberrant accumulation of either mutant or wild type Tar DNA-binding protein of 43 kDa (TDP-43) has been found in both fALS and sALS, thus accounting for a common mechanism involving aberrant RNA processing and glutamate excitotoxicity [1, 4–10].

SMA is the most common inherited motoneuron disease and the main genetic cause of newborn mortality. Like ALS, SMA is characterized by the loss of spinal and bulbar motoneurons. In contrast to the multifactorial origin of ALS, this disease is unambiguously caused by the recessive mutations or deletion of the Survival Motor Neuron-1 gene (SMN1) [11–13].

A number of animal models have been developed attempting to recapitulate at least some of the genetic, anatomical, and functional defects observed in the human ALS and SMA [10, 14–17]. These models have also been used for testing the efficacy of different repairing strategies such as rehabilitation, pharmacological, genetic, or cell-based approaches [10, 16–26].

SC injury (SCI) or nerve damage could also result in severe loss of grey matter neurons, including motoneurons [27, 28]. The mechanism of cell loss after contusion injury is complex: the mechanical damage of SC tissue (primary injury) destroys many local neurons, but it is followed by a secondary injury that kills a larger neuronal and glial population because of several pathological phenomena, including inflammation or vascular damage [29].

Although the described neurodegenerative or traumatic SC diseases are different in their etiology and pathogenesis, they share a common outcome characterized by the death of lower motoneurons. Regardless of the pathological reason for motoneuron loss, several studies have investigated the possibility of repairing the motoneuron-depleted SC by using different repairing strategies. These studies have used several animal models of selective motoneuron depletion [30, 31].

In the present paper, we performed a comprehensive review of the literature about the use of rodent models of neurotoxic spinal motoneuron degeneration, with a focus on two models obtained by intramuscular injection of volkensin or cholera toxin-B saporin (CTB-Sap). In particular, the experimental applications of these models to mimic neurodegenerative diseases, to dissect the molecular mechanisms of neuroplastic changes underlying the functional recovery after motoneuron loss, and to evaluate the effectiveness of several strategies of neural repair are extensively discussed in comparison to the other available preclinical models of disease.

## 2. Rodent Neurotoxic Spinal Cord Lesion Models

The first evidences about the effects of neurotoxins on motoneurons were provided as early as fifty years ago, with some studies showing the effects of tetanus and botulinum toxins on spinal motoneurons [32–34]. Afterwards, functional neuroanatomy studies have relied on the effects of lesions to investigate the function of neural systems, and a large variety of neurotoxins has been used to destroy specific cell populations. For instance, excitotoxins such as kainic acids [35, 36] or monoamine toxins including 6-hydroxydopamine [37] have been used to produce selective lesions based on the neurotransmitter specificity, but these compounds have shown incomplete anatomical and cell-type specificity. A substantial improvement of these methods of “molecular neurosurgery” has been provided by the development of axonally transported toxins such as lectins [38–41], immunotoxins [42–45], tracer-toxins, and neuropeptide-conjugated toxins [42]. When injected into the target region, these toxins are captured by axon terminals and retrogradely transported towards the cell body, thus causing cell death by ribosome inactivation and apoptosis. Plant derived lectins are anatomically but not cell-type selective, being able to kill any neuron projecting to the injection site, by suicide retrograde transport [39–41, 46, 47]. This term refers to the uptake and axonal transport of toxins by neurons projecting to the injection site, thus causing a selective lesion based on the specific neural connection rather than cell phenotype [31, 42, 47, 48]. Conversely, immunotoxins as well as tracer- or neuropeptide-conjugated toxins are both anatomically and cell-type selective, since they are internalized by cells after specific chemical binding [30, 42, 49].

A large number of plant derived neurotoxic proteins have been isolated and characterized [50], thus showing their ability to damage eukaryotic cells by acting on ribosome and catalytically disrupting the elongation step of protein synthesis [51, 52]. These ribosome-inactivating proteins (RIPs) include ricin (from* Ricinus communis*), abrin (from* Abrus precatorius*), modeccin (from* Adenia digitata*), and volkensin (from* Adenia volkensii*) [38, 39, 50, 52]. All these RIPs are axonally transported by peripheral nerves but, among these, modeccin and volkensin are more efficient to kill neurons of the central nervous system (CNS) by suicide transport [40–42, 46, 53]. Among the above described RIPs, volkensin [39] appeared to be the most toxic on CNS neurons and it has been the most frequently used to create animal models of spinal motoneuron degeneration. As early as in 1992, Nógrádi and Vrbová used volkensin with the aim of creating a reliable model of motoneuron degeneration [31]. Similar long-term effects of volkensin on the SC results were shown by Leanza and Stanzani (1998) after intramuscular injection of 2.0 ng of this RIP in newborn rats [54]. These authors have reported an extensive and long-lasting depletion of spinal motoneurons (about 90%) as measured at either two or eight months after the lesion. Afterwards, this rodent model was used, also by our research group, either as recipients in experimental approaches of transplant-induced regeneration (see Section 4) [55–57] or as models for testing the intrinsic potential for spontaneous regeneration (see Section 3) [58].

A substantial improvement of neurotoxic lesion protocols came from the development of targeted RIPs by conjugation with a specific carrier, such as an antibody, a neuropeptide, or a retrograde tracer [30, 42, 44, 45, 49, 50, 59]. Saporin, an RIP from* Saponaria officinalis* [50], is the most used toxin to prepare targeted neurotoxins. Cholera toxin is the bacterial protein toxin of* Vibrio cholerae*. It is composed of a catalytically active A subunit linked with a B subunit. The latter is responsible for the specific binding to the GM1 membrane receptor, internalization, and retrograde transport [60, 61]. Given these properties, cholera toxin-B subunit could be used either as a retrograde tracer [30, 62] or as a targeted neurotoxin after conjugation with saporin [30]. A number of* in vivo* experiments have used cholera toxin-B saporin (CTB-Sap) and demonstrated its effectiveness in removing any neuron expressing GM1 ganglioside [30, 49, 63–65]. Recently, our group has developed a mouse model of lumbar SC motoneuron degeneration by injection of CTB-Sap into the gastrocnemius muscle. The toxin has been injected into the medial and lateral gastrocnemius muscles at a dose of 3.0 *μ*g/muscle and caused a partial depletion of lumbar motoneuron (25–30%), accompanied by an evident impairment of the hindlimb motor function [66]. Given the moderate severity of the lesion, this model is suitable for evaluating the spontaneous recovery of locomotion and the underlying SC plastic changes, such as neurogenesis [66] or synaptic plasticity (see Section 3) [66–69].

## 3. Mechanisms of Spinal Cord Plasticity in Models of Motoneuron Disease

Several evidences have demonstrated that adult mammals could achieve a significant range of spontaneous sensory-motor recovery after injury or disease, by means of various forms of neuroplasticity. This plasticity includes the recruitment of neural precursor cells (NPCs) and the formation of new pathways as well as synaptic plasticity, within the affected tissue and/or in sensory and supraspinal pathways [70–73]. However, this spontaneous plastic potential is inadequate for allowing complete regeneration and recovery of function, but some therapeutic interventions are able to recruit and potentiate this intrinsic capacity, thus producing a better outcome. Since it has been found that SC plasticity is activity-dependent [74], a number of studies have demonstrated the effectiveness of exercise training and other methods of “spinal learning” in both animal models and human SCI patients [70, 75–77]. Some information is also available about plastic changes occurring in neurodegenerative diseases and, in particular, in motoneuron disease. It is known, for instance, that plastic changes could occur in Parkinson's disease [78] as well as in the respiratory system and brain of ALS patients [79–81], but the beneficial effect of exercise training is still controversial [82, 83]. Given the progressive nature of these diseases, it is obvious that any compensatory change will ultimately be ineffective. Despite these limitations, a better understanding of the plastic phenomena occurring in animal models of motoneuron disease would help in elucidating the molecular mechanisms of diseases and finding new putative targets for therapy. Anatomical rearrangement and functional compensatory changes in spinal and supraspinal circuitry have been reported in rodent models of neuronal degeneration induced by nerve crushing [84, 85].

The previously described murine model of selective CTB-Sap induced motoneuron depletion developed in our laboratory has been deeply characterized to evaluate its capacity for spontaneous sensory-motor recovery. Noteworthy, a relevant increase of motor performance measured at the grid walk or rotarod test has been observed as early as one month after toxin injection, despite a permanent though moderate motoneuron removal [66, 68, 69]. The cellular and molecular mechanisms underlying this remarkable functional recovery have been studied, including the activation of endogenous NPCs [66], the spontaneous events of synaptic plasticity [66–69], and the expression and functional roles of neurotrophic factors [67] and/or other molecular factors including cell fate determinants [66–68] and TDP-43 [69].

### 3.1. Neurogenesis

NPCs proliferation and differentiation take place spontaneously in the adult mammals only in the subventricular zone and hippocampus [86, 87]. However, multipotent NPCs could be isolated from the entire adult CNS, including the SC [88–90]. Several experiments have demonstrated that these cells could be mobilized after SCI but, unfortunately, they only generate migratory cells that differentiate to astrocytes and participate in scar formation [89, 91, 92]. Notably, astrocyte activation could also be caused by a selective neurotoxic neuron removal by volkensin suicide transport in either brain or SC [31, 93]. Moreover, a significant amount of cell proliferation and increase of GFAP-positive astrocytes have been found in the SC ventral horn, after selective motoneuron removal by intramuscular injection of CTB-Sap [66]. Glial reaction is a classical response to CNS tissue damage, which generally also involves glial cells themselves and induces a series of events that amplifies and maintains glial activation [94, 95]. Therefore, the glial reaction observed after selective neuronal loss, with the absence of severe tissue damage and inflammation [96], could have different origin as well as different consequences on regenerative processes.

**Figure 1:**
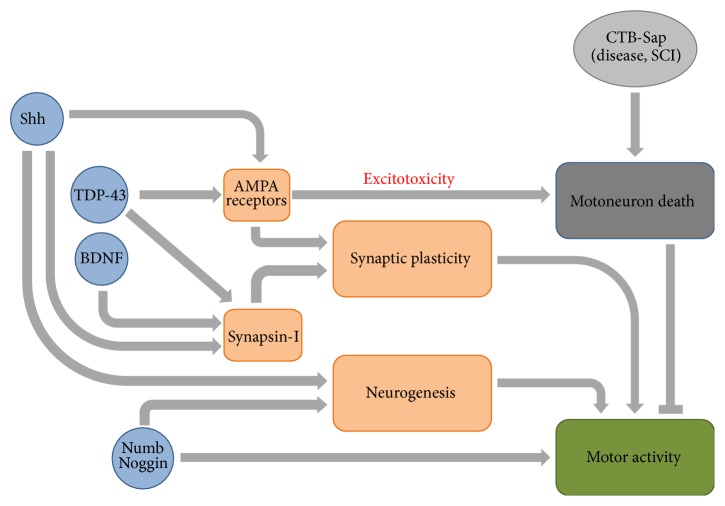
Proposed model of spontaneous SC plasticity after motoneuron degeneration.

Intrinsic and extrinsic molecular factors regulating adult neurogenesis have been widely explored [86, 97]. Sonic hedgehog (Shh) is a secreted glycoprotein promoting NPCs proliferation and differentiation to neurons and oligodendrocytes, during both development and adulthood [98, 99]. The Notch-1 pathway and its inhibitor Numb are also involved in the regulation of NPCs proliferation, cell fate determination, dendritic morphology, and axon guidance in embryonic and adult CNS [100–103], including SC [104, 105]. Noggin is a secreted glycoprotein responsible for neural induction during development, by acting as an inhibitor of bone morphogenetic proteins [106]. As shown by Chen and colleagues (2005), Shh, Notch-1, and Numb expression are increased in the SC after compression injury [107]. However, unlike their embryonic counterparts, NPCs are unable to generate neurons in the adult SC. Recently, some experiments have been performed to investigate the expression and the functional role of Shh, Notch-1, Numb, and Noggin on the murine model of CTB-Sap induced motoneuron depletion [66, 68]. In contrast to those observed in SCI models, Shh and Numb expressions appear transiently decreased after motoneuron removal and then recovered in association with the spontaneous functional recovery, whereas Noggin expression progressively increases [66, 68]. The reasons for the discrepancy between mechanical and neurotoxic lesion models are elusive but some explanations could be proposed. For instance, mechanical damage affects several neuronal and glial populations, whereas the described neurotoxic lesion selectively kills motoneurons in spatially restricted regions. Moreover, ependymal cells undergo a robust proliferation immediately after a mechanical injury [89, 108], whereas they seem unresponsive in the CTB-Sap model [66] (see Figure 1).

Interestingly, a pattern of NPCs proliferation and reactive gliosis closely resembling that found in CTB-Sap models, with no evidence of neurogenesis, was found in transgenic mouse models of ALS expressing the mutated human SOD1 gene [109, 110]. Unfortunately, further information concerning these endogenous repairing potentials of ALS affected SC is still lacking, and the results provided by neurotoxic models are therefore of great importance. However, these processes need to be further clarified because they denote the importance of the role of environmental cues on the behavior of spinal NPCs. It is also likely that an experimental approach aimed at artificially modifying Shh, Numb, and Noggin signaling into the SC could stimulate NPCs proliferation, reduce glial reaction, and probably drive cell differentiation towards neuronal phenotype.

### 3.2. Synaptic Plasticity

Another process promoting the functional restoration consists of the reorganization of spinal, supraspinal, and sensory pathways by mechanisms involving activity-dependent synaptic plasticity [71, 74, 111]. As previously described, a significant amount of spontaneous locomotor recovery is possible in rodent models of both SCI and motoneuron disease and could be driven, at least partially, by mechanisms of synaptic plasticity [66–69, 112, 113].

The molecular feature of synaptic plasticity has been extensively studied in the hippocampus, as it represents the principal mechanism underlying learning and memory. In fact, it is known that long-term modifications of synaptic efficacy are regulated presynaptically by the expression and phosphorylation of various synaptic vesicle proteins including synapsin-I [114–116] and postsynaptically by changes in the expression and trafficking of glutamate receptors. In particular, alpha-amino-3-hydroxy-5-methyl-4-isoxazolepropionic acid (AMPA) ionotropic glutamate receptors are fundamental for cortical and hippocampal synaptic plasticity [117–120]. The emerging role of astrocytes and their expression of connexins in the modulation of synaptic strength are also noteworthy [121, 122].

A fundamental role in modulating both pre- and postsynaptic changes is exerted by brain-derived neurotrophic factor (BDNF) [123–125]. In fact, synapsin-I is considered as a downstream effector of BDNF [123, 125]. Moreover, it seems clear that the activity-dependent release of BDNF could regulate the synthesis and synaptic delivery of AMPA receptors in different brain areas [126, 127] and, conversely, the glutamate receptor activity could modulate BDNF release [128, 129]. Interestingly, several authors have shown that such mechanisms could take place also in the intact and lesioned SC [112, 113, 130], as well as in the mouse model of CTB-Sap induced motoneuron loss developed in our laboratory. In particular, we have found that the spontaneous recovery of locomotion observed in the motoneuron-depleted mice is linked to the expression levels of both synapsin-I and AMPA receptors [66–68]. Moreover, this model has confirmed the described role of BDNF [67] and has also provided evidence about novel functional roles of Shh, Numb, and Noggin that, in addition to the traditional role as cell fate determinants, could also participate in modulating synaptic plasticity and functional recovery [66–69] (see Figure 1).

Information about the occurrence of synaptic plasticity in patients or animal models of ALS is poor. However, it is noteworthy that the expression of synaptic vesicle proteins is significantly decreased in the SC ventral horn of ALS patients [131], thus again confirming that CTB-Sap models could be interesting research tools for research in motoneuron disease.

### 3.3. The Emerging Role of TDP-43

TDP-43 is a nuclear DNA/RNA-binding protein encoded by a highly conserved gene and involved in mRNA processing [132, 133]. Recently, TDP-43 was found in the cytoplasmic protein aggregates observed in some neurons of patients affected by ALS [6, 133]. Therefore, increasing attention has been devoted to the toxic effects of mutant TDP-43 on motoneurons but, more recently, it is becoming likely that some of these effects could depend on the loss of function of the normal TDP-43 [5, 7, 133, 134]. In addition to the described classical role, TDP-43 could be involved in apoptosis, microRNA biogenesis, and cell proliferation [132]. Notably, TDP-43 has been found in the dendrites, where it could affect local RNA translation in an activity-dependent manner [135, 136]. Moreover, TDP-43 is crucial for synaptic formation and plasticity, as well as for locomotion in* Drosophila* [134, 137, 138].

It has been recently shown in our model of motoneuron loss that synapsin-I expression is linked to that of TDP-43 and that the latter correlates with the expression of AMPA receptor subunits GluR1, GluR2, and GluR4 [69]. This association is interesting. As mentioned above, synaptic plasticity is modulated by AMPA receptor trafficking and in particular by the regulation of Ca^2+^-permeable AMPA receptors [117–119]. The ion permeation is linked to the amount of Q/R-unedited GluR2 subunits included into the AMPA channels. Therefore, given that TDP-43 is likely involved in the Q/R-editing of GluR2 subunits, one of the proposed mechanisms of motoneuron death in ALS is the glutamate toxicity caused by the aberrant increase of unedited GluR2 subunits [5, 8]. Similar processes could take place in the CTB-Sap SC lesion model and, interestingly, the same events could affect synaptic plasticity in this model. Unlike the functional linkage between AMPA receptors and TDP-43, the association between synapsin-I and TDP-43 is absolutely novel and suggests a model where TDP-43 could affect synaptic strength by modulating the expression of both synapsin-I and AMPA receptors [69] (see Figure 1). This hypothesis is supported by other evidences that TDP-43 is present at synapses and controls the local synthesis of synaptic proteins [135, 139]. Other recent findings have shown that the lack of TDP-43 could affect synapses and cause locomotor deficits in* Drosophila* [134, 137].

Given the increasing interest in mouse models of TDP-43 gain or loss of function as models of neurodegenerative diseases, including ALS [10, 16, 140], is likely that the elucidation of the physiological role of TDP-43 in the SC would provide an important contribution.

## 4. Repairing Strategies

To date, neurodegenerative disorders such as ALS and SMA do not benefit from any effective therapy. Riluzole represents the only approved therapy for ALS, but its effects consist in prolonging survival and delaying the use of supportive care by a few months [141]. As previously discussed, the adult SC is capable of a significant amount of spontaneous functional restoration, and this is particularly evident in rodent models of SC injury or disease [66, 68, 71, 74, 111]. Although this capacity is not enough to allow full recovery, it is anyway encouraging because the elucidation of the underlying cellular and molecular mechanisms would provide novel therapeutic tools and targets, thus improving the expected clinical outcomes. As the spontaneous functional recovery could be driven by the recruitment of NPCs, regeneration of damaged neurons, and events of synaptic plasticity occurring within the spared circuitries, the improvement of these processes by external interventions would represent effective therapeutic strategies. Several preclinical studies have shown that cell-based therapies could also be promising. However, further studies employing representative preclinical models, as well as the design of clinical trials, are mandatory to make this increasing knowledge available for translational applications.

### 4.1. Non-Cell-Based Therapies

The activity-dependent nature of plastic changes within the SC [71, 74] has suggested the possibility that the damaged SC could be retrained in an attempt to modify the activity of the spared circuitries and compensate for the partial loss of neurons and connections [142]. Several animal models of SCI have been used so far to test this hypothesis. Locomotor training has proven to be beneficial in spinalized animals [76, 112, 113], by mechanisms of activity-dependent BDNF-induced synaptic plasticity [112, 113, 130, 143]. Significant clinical improvement could also be achieved by human SCI patients as a result of locomotor training [70, 75]. The importance of plastic changes in motoneuron diseases needs further investigations and the data provided by neurotoxic models would also be helpful as previously discussed. Few studies have investigated the therapeutic value of exercise training in either human patients or animal models of motoneuron disease and produced controversial findings. A couple of studies involving SOD1 mouse models demonstrated that a moderate exercise could produce neuroprotective effects on motoneurons, although the impact on the life span is controversial [144–146]. Moreover, the beneficial effects seem to be dependent on the type of physical exercise [18]. Similar results have been provided by a small number of studies involving human patients [82, 83], thus indicating that further studies are needed to clarify the relationships among neuronal activity, motoneuron vulnerability, and neuroprotection. In this respect, important insights have been provided by the previously described CTB-Sap SC lesion model (see Section 3) [66–69], but some of them require further investigation and clinical trials. In particular, the role of neurotrophins and other growth factors has been confirmed in different animal models including the CTB-Sap lesioned and the other established animal models of disease [17, 147]. However, human trials showed inconsistent or negative effects of growth factors due to different reasons such as bioavailability, poor penetration through the blood-brain barrier, and inadequate or excessive dosing.

Other studies for effective treatments have focused on the neuromuscular junction and the role of the skeletal muscle as source of chemical and cellular cues sustaining neuronal survival, axonal growth, and synaptic connections, such as trophic support or the role on Nogo-A [19]. The CTB-Sap model could help in investigating this aspect without unwanted environmental cues, which are normally present in the genetic models of ALS or SMA.

### 4.2. Cell Therapy

Cell transplantation was one of the first repairing approaches used in models of SC injury and disease. Transplantation of fetal motoneurons was successfully used in models of motoneuron loss induced by nerve crushing [148], kainic acid [149], or volkensin [55–57, 150] and demonstrated that the grafts were able to survive and develop as functionally active mature motoneurons [55–57, 150], although their capacity of muscle reinnervation was limited. More recently, cell-based strategies have relied on the potential beneficial effects of stem cells such as embryonic, neural, mesenchymal, and induced pluripotent stem cells [19–26, 151–153]. A number of preclinical studies have proven that stem cell therapy is able to delay the disease progression, rescue motoneuron function, and extend survival in animal models of ALS or SMA. Multiple mechanisms are responsible for these beneficial effects. It is obvious, for instance, that replacement of lost motoneurons is an important goal in repairing strategies, but some limitations still occur as previously described, including integration into the host tissue and reinnervation. Moreover, resident as well as grafted neurons could be susceptible to degeneration if exposed to a toxic microenvironment like that present within the diseased neural tissue. Transplantation of cells including different stem cell types could provide trophic support, remove toxic cues, and exert immunomodulatory effects, which ultimately could result in neuroprotection for motoneurons [19–26, 151–153]. The use of a neurotoxic model, where motoneuron depletion is not accompanied by a chronic disease state or toxic environment, could offer a different point of view for elucidating the beneficial effects of cell-based therapies. A number of stem cell clinical trials [19, 154] have shown that some cell-based protocols could be safe and produce promising though modest effects. Regarding the cell source, mesenchymal stem cells could be easily obtained from patients and are considered suitable for autologous transplantation. Interestingly, induced pluripotent stem cells represent a novel source for autologous stem cells. They can be obtained by reprogramming somatic cells without viral methods and differentiated towards multiple phenotypes [19–26, 151–154]. However, to achieve effective cell-based therapies suitable for clinical application, several issues should be addressed, including the optimization of delivery protocols (route of administration, dose) and the better elucidation of the graft-host interaction. The ideal route of administration should produce the best therapeutic effects with the minimal invasiveness. Intrathecal or intravenous administration could represent effective approaches, because they ensure the widespread distribution of cells, which is ideal when degeneration is not limited to a small area. However, cells must be able to penetrate the blood-brain barrier and migrate correctly towards the affected areas. Again, several preclinical studies are needed, by using different animal models, to address these important goals.

### 4.3. Recruitment of Endogenous Neurogenesis

As previously discussed, NPCs proliferation occurs in different animal models of motoneuron loss, including neurotoxic and ALS models, but external interventions are needed to potentiate this capacity and drive NPCs differentiation towards the neuronal phenotype [66, 109, 110]. Bambakidis and colleagues (2003) have treated SC lesioned rats with Shh and provided evidence of increased NPCs proliferation and their differentiation as oligodendrocytes and neurons [73, 155]. In addition, Shh promotes survival and exerts neuroprotective effects on CNS neurons including motoneurons [156, 157]. A recent study showed that G93A mouse model of ALS produced spontaneous NPCs proliferation within SC lamina X, which was increased by lithium administration. Moreover, lithium-treated animals showed increased neuronal differentiation and attenuation of disease progression [110]. Another growth factor not only stimulating neurogenesis but also promoting neuronal survival, migration, and axon guidance in ALS models as well as protection of motoneurons against excitotoxicity is Vascular Endothelial Growth Factor (VEGF) [158, 159]. Beneficial effects of many other growth factors and morphogens, as well as hormones, on SC repair have been published by several authors. Axonal growth and other plastic changes could be promoted, for instance, by Noggin and BDNF [113, 160–162], whereas testosterone treatment has proven to exert neuroprotective effects on motoneurons in CTB-Sap lesion models, by preventing dendritic atrophy after removal of surrounding motoneuron [63, 65].

## 5. Concluding Remarks

Further studies are needed to better understand the mechanism of neurodegeneration as well as develop effective methods of therapy and rehabilitation. In this respect, although a large number of studies will be obviously conducted on mouse models of ALS and SMA, the above-described neurotoxic models of motoneuron degeneration will certainly be useful as well. In fact, these models are easy to be produced and characterized. Moreover, motoneuron-depleted SC is a simple and powerful tool for cell transplantation and for testing plastic changes and the consequent functional outcome. Despite the difference between neurotoxic and genetic rodent models, the described similar effects on neurogenesis and the involvement of TDP-43 and the multiple roles of neurotrophins and morphogens would open a number of novel research pathways aimed at the dissection of pathogenesis and selection of new therapeutic targets and tools for the treatment of motoneuron diseases.
